# Bioinformatics Commons: The Cornerstone of Life and Health Sciences

**DOI:** 10.1016/j.gpb.2018.09.001

**Published:** 2018-09-28

**Authors:** Zhang Zhang, Yu Xue, Fangqing Zhao

**Affiliations:** 1 BIG Data Center and CAS Key Laboratory of Genome Sciences & Information, Beijing Institute of Genomics, Chinese Academy of Sciences, Beijing 100101, China; 2 Department of Bioinformatics & Systems Biology, MOE Key Laboratory of Molecular Biophysics, College of Life Science and Technology and the Collaborative Innovation Center for Biomedical Engineering, Huazhong University of Science and Technology, Wuhan 430074, China; 3 Beijing Institutes of Life Science, Chinese Academy of Sciences, Beijing 100101, China

Bioinformatics, an interdisciplinary field that combines biology, mathematics, computer science, medicine, and health science, to integrate, analyze, and interpret biological data, is now becoming increasingly data-intensive. To dig out the treasure from big data powered by high-throughput sequencing technologies, it is highly dependent on ***Bioinformatics Commons*** that involves a variety of fundamental resources, including databases, web servers, algorithms, methods, software tools, ontologies, and standards. To be short, *Bioinformatics Commons* has become the cornerstone of life and health sciences, facilitating and accelerating the translation of big data into big discoveries.

Over years, a number of resources in *Bioinformatics Commons* have been developed and publicly released, with the aim to ease users to explore the relationship between genotype and phenotype and improve our understanding of genetic basis for human diseases. Hence, it is pivotal to raise the general awareness of the significance of *Bioinformatics Commons* in knowledge discovery. Toward this end, as a consequence, we propose to set up the Special Issue on *Bioinformatics Commons* this year. In this issue, *Bioinformatics Commons* (I), we included 9 resources (8 databases and 1 web server; Table 1), covering a diversity of topics on DNA methylation, post-translational modifications, circular RNAs, genetic variant annotation, association studies, cancer, and other diseases, as well as plant defense responses. All databases in this issue have been added to Database Commons (http://bigd.big.ac.cn/databasecommons/), a catalog of global biological databases, in BIG Data Center [10]. Articles on algorithms, methods, and application notes of software tools will be included in *Bioinformatics Commons* (II) in the next issue.

**Table 1:** **Resources in the Special Issue on Bioinformatics Commons**

**Name**	**Short description**	**Link**	**Ref.**
CIRCpedia	A database of circular RNAs	http://www.picb.ac.cn/rnomics/circpedia	[1]

HeteroMeth	A database of single-cell DNA methylomes	http://qianlab.genetics.ac.cn/HeteroMeth	[2]

PTMD	A database of human disease-associated post-translational modifications	http://ptmd.biocuckoo.org	[3]

GAAD	An autoimmune disease association database	http://gaad.medgenius.info	[4]

CCGD-ESCC	A comprehensive database for genetic variants associated with esophageal squamous cell carcinoma in Chinese population	http://db.cbi.pku.edu.cn/ccgd/ESCCdb	[5]

HCCDB	A database of hepatocellular carcinoma expression atlas	http://lifeome.net/database/hccdb	[6]

TSNAdb	A tumor-specific neoantigen database	http://biopharm.zju.edu.cn/tsnadb	[7]

PlaD	A transcriptomics database for plant defense responses to pathogens	http://systbio.cau.edu.cn/plad/index.php	[8]

DeepNitro	A web server for predicting protein nitration and nitrosylation sites	http://deepnitro.renlab.org	[9]

*Note*: All databases listed here have been added to the Database Commons at http://bigd.big.ac.cn/databasecommons/.

Clearly, this Special Issue on *Bioinformatics Commons* exhibits several features. First, it is noted that extensive efforts, in coincidence with precision medicine, have been made related to human diseases through literature curation [3], potentially providing valuable clues into precision healthcare and treatment on a variety of diseases, especially autoimmune disease [4], esophageal squamous cell carcinoma [5], hepatocellular carcinoma [6], and collecting tumor-specific neoantigens as potential targets for cancer immunotherapy [7]. Second, several cutting-edge research topics are involved. For instance, CIRCpedia [1] features integration of comprehensive circular RNAs and their annotations, HeteroMeth [2] features single-cell DNA methylomes, and PlaD features transcriptomic characterization of plant defense responses to pathogens [8]. Third, advanced information technology has been applied in bioinformatics, as exemplified by DeepNitro [9] that features adoption of deep learning technologies for prediction of protein nitration and nitrosylation sites. Taken together, *Bioinformatics Commons* provide valuable resources in aid of big data integration, translation, and interpretation, and accordingly are of broad utility in advancing life and health sciences. Thus, the Special Issue on *Bioinformatics Commons* is expected to be issued annually in *Genomics, Proteomics and Bioinformatics* and we look forward to your submissions for consideration of future publication in this Special Issue.

## Competing interests

The authors have declared no competing interests.
